# Spatial Dynamics of a Phlebotomine Sand Flies Population in Response to Climatic Conditions in Bushehr Province of Iran

**DOI:** 10.5334/aogh.30

**Published:** 2019-04-23

**Authors:** Zahra Zarei Cheghabalaki, Dariush Yarahmadi, Mostafa Karampour, Aliakbar Shamsipour

**Affiliations:** 1 Climatology, Lorestan University, Lorestan, IR; 2 Climatology, Tehran University, Tehran, IR

## Abstract

**Background:**

Phlebotomine (Ph.) sand flies are the vectors of different types of leishmaniasis and arboviruses to humans. Variations in climate conditions could lead to changes in the dynamics of zoonotic cutaneous leishmaniasis (ZCL) and its agents, such as Ph. sand flies and reservoirs.

**Objective:**

The aim of this study was to analyze the spatial relationship among climatic factors and phlebotomine sand flies in abundance in Bushehr Province of Iran.

**Materials and Methods:**

In this study, six village sites in Bushehr Province were selected for collecting Ph. sand flies. All-night landing catches of Ph. sand flies occurred between January 15, 2016, and December 15, 2016. Two types of climatic data were used: satellite-based data included daytime and nighttime land surface temperature and the normalized deference vegetation index, and station-based data included annual rainfall and annual mean air temperature and solar radiation (W/M2). Spatial correlation analysis and multivariate ordinary least square regression was used to detect the spatial association between caught Ph. sand flies and climatic factors in a 0.95 confidence level (p = 0.05).

**Results:**

The results of the spatial correlation matrix indicated thermal factors, such as mean and maximum air temperature, instantaneous daytime and nighttime land surface temperatures, and radiation, show a direct and significant spatial correlation with the number of sand flies caught. The annual frequency of sand flies in the region showed the highest direct spatial relationship with the annual maximum air temperature (r = 0.91). Environmental moisture factors, such as precipitation and the vegetation index of the region, have spatial correlations of 0.63 and 0.44 with the frequency of the annual caught sand flies, which are significant at the confidence level of 0.95. The results of the multivariable model for estimating the frequency of the caught sand flies indicate environmental estimators, including mean air temperature, rainfall, radiation, and vegetation index, in a linear estimation model can explain the 0.85 spatial variation of the caught sand flies population.

This study aimed to detect spatial correlations between the sand fly population and climatic factors. The results showed climatic factors were the most important controllers of the sand fly population in the interannual scale. If sand fly control programs are conducted in accordance with the climatic conditions of the area, the population of this carrier in Bushehr Province can be controlled and the incidence of disease significantly reduced.

## Introduction

Leishmaniasis is one of the most important vector-borne diseases [1]. There are three main forms of leishmaniasis: zoonotic cutaneous leishmaniasis, anthroponotic cutaneous leishmaniasis, and visceral leishmaniasis. All three are public health problems in Iran and its neighboring countries, such as Afghanistan and Pakistan [2]. Cutaneous and visceral leishmaniasis (with a leishmania agent) are endemic in many parts of the world, including Iran, and are transmitted by the bites of various species of Phlebotomine sand flies [3]. These insects have been studied with considerable attention and have achieved major global importance because of their role in the transmission of pathogens responsible for bartonellosis, arboviroses and leishmaniasis [26,27,28].

Sand flies (Diptera: Psychodidae and Phlebotominae) belong to the genera Phlebotomus and Lutzomyia and are the vectors of leishmaniasis. Phlebotomine sand flies are found mostly in tropical and subtropical areas, and a limited number are found in temperate regions [5]. The subfamily Phlebotominae includes about 900 species, but no more than 70 have been implicated in leishmaniasis transmission [25]. About 30 species of sand flies are proven vectors of at least 20 Leishmania species [4]. In Iran, there are 52 reported species of sandflies belonging to the subgenera Adlerius, Euphlebotomus, Larrousius, Paraphlebotomus, Phlebotomus, Synphlebotomus, Grassomyia, Parrotomyia, Parvidens, Rondanomyia, Sergentomyia, and Sintonius2.

Phlebotomus papatasi and P. salehi are the main and secondary vectors of zoonotic cutaneous leishmaniasis (ZCL) caused by Leishmania major in Iran. Leishmania major is also isolated from P. caucasicus group and P. ansarii in rodent burrows [1,6]. In Iran, according to surveying on vectors of ZVL in important endemic foci of the disease, four sand flies species belonging to Larroussius subgenus, including Phlebotomus (Larroussius) kandelakii, P. (Larr.) perfiliewi transcaucasicus, P. (Larr.) keshishiani, and P. (Larr.) major, and one species of Paraphlebotomus subgenus, including P. (Para.) alexandri, have been observed naturally infected with promastigote due to Leishmania infantum [7,8].

The first comprehensive entomological study on sand flies of Iran was done by Mesgali (1960), who reported 12 species belonging to the genus Phlebotomus and 11 species belonging to the genus Sergentomyia [9]. Later, Javadian and Mesghali (1974) reported 42 species of Phlebotomine sand flies in Iran [10]. More recently, it has been shown that the fauna of Iran includes 44 confirmed species and 10 unconfirmed, as reported by the latest Iranian Phlebotomine sand fly faunistic studies [11,12,13,14,15,16]. Kassiri et al. (2000) have proposed a checklist of Iran sand flies that includes 54 species. In Iran and in the Old World [12], P. papatasi is recognized as the main vector of leishmaniasis to humans [13,15].

The spatiotemporal variation of environmental factors, such as temperature, precipitation, and humidity, affect the biology and ecology of vectors and intermediate hosts and, consequently, the risk of disease transmission [17]. These climatic factors affect the digestion, metabolic processes, and developmental times of sand flies [18]. Ambient temperature also affects the developmental rates of the immature stages, survival of pre-imaginal stages, and longevity of the adult phlebotomine sand flies [19,20].

Leishmaniasis is strongly affected by ecological factors. Because the leishmania parasites are transmitted through the bites of infected female phlebotomies and flies, the epidemiology of leishmaniasis depends on the characteristics of the parasite species, the local ecological characteristics of the transmission sites, current and past exposure of the human population to the parasite, and human behavior. Current evidence suggests geographical factors also have a direct influence on the epidemiology of vector-borne diseases [20].

Iran is among seven countries with a high incidence of CL [21]. CL is endemic among half of the 31 provinces of Iran [22]. The geography and climate conditions of Iran are conducive to the growth and proliferation of the sand flies transmitting CL [23]. The number of CL cases has progressively increased from 11,505 to 26,824 from 2001 to 2008 in Iran [24].

According to Kamhawi (2006), about 350 million people are at risk of contracting leishmaniasis and some 2 million new cases occur each year, mostly in developing countries [29]. Therefore, the main object of this study is to analyze the spatial association among Ph. sand fly population dynamics and climatic factors.

## Materials and Methods

### Study Area

**Figure 1:**
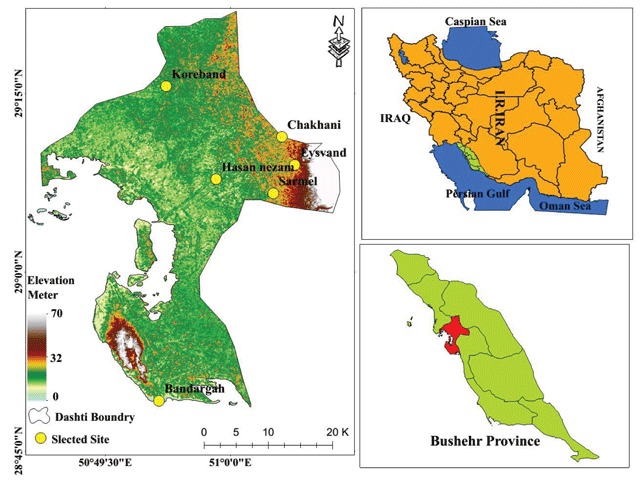
Study area and the location of selected sites for catching Ph. sand flies.

Bushehr county was selected as the site of study. As can be seen in Figure 1, it is centrally located. Bushehr county is one of nine counties in Bushehr province of Iran. It has an area of 5,008 km^2^. At the 2006 census, the county’s population was 71,285 from 15,465 families. The capital of the county is Khormuj. The county is subdivided into three districts: the central district, Kaki district, and Shonbeh and Tasuj district. Bushehr County is bounded by the Persian Gulf to the west, Tangestan County to the north and west, Dashtestan County to the northeast, Dayyer County and Kangan County to the south, Jam County to the southeast, and Firuzabad County to the east. Bushehr County has a 25 km coastline along the Persian Gulf, with a climate that is mostly arid or semiarid. The highest point in the county is Mount Beyrami at 1,950 m.

### Environmental Data

In the present study, two types of climatic data were used (Table 1). Satellite-based data included daytime land surface temperature (DLST; C), nighttime land surface temperature (NLST; C), the normalized deference vegetation index (NDVI), monthly mean accumulated rainfall (mm), and monthly mean 2-m air temperature (C) from January 2000 to December 2016 (17-year average).

**Table 1:** Environmental Data Used to Explain Spatiotemporal Dynamics of Phlebotomus Population in Response Climate Conditions.

Data Type	Product	Time	Description	Time duration	Format	Source

Land Surface Temperature	MOD11C 3	Daytime	Annual	Jan 2000–Dec 2016	.HDF	MODIS Website
		Nighttime				
2 m Above Land Surface Air Temperature	ECMWF	3.5 PM IR	Annual	Jan 2000–Dec 2016	.NC	http://www.ecmwf.int/
Rainfall	ECMWF	3.5 PM IR	Annual	Jan 2000–Dec 2016	.NC	http://www.ecmwf.int/
Radiation	ECMWF	3.5 PM IR	Annual	Jan 2000–Dec 2016	.NC	http://www.ecmwf.int/
Vegetation indexes	MOD 13A3	Monthly	Annual	Jan 2000–Dec 2016	.HDF	MODIS Website

ECMWF, European Centre for Medium-Range Weather Forecasts; HDF, Hierarchical Data Format; MODIS, Moderate Resolution Imaging Spectroradiometer; LST, land surface temperature; DLST, daytime LST; NLST, nighttime LST; NC, Net CDF (Network Common Data Format).

*Normalized Deference Vegetation Index.* Initially, different ratios and normalized indices were determined based on a combination of visible and near-infrared wavelength, as suggested by scientists. NDVI was obtained with the following expression:

1 NDVI = \frac{{\left({NIR - VISr} \right)}}{{\left({NIR + VISr} \right)}}

Where NDVI stands for normalized difference vegetation index, NIR stands for near-infrared radiation, and VISr stands for visible red spectrum. NDVI values range from –1 (usually water) to +1 (strongest vegetative growth). The amount of reflectance in the NIR range (λ = 700–1300nm) and in the VISr range (λ = 550–700nm) is determined by the optical properties of the leaf tissues: their cellular structure and the air-cell wall-protoplasm-chloroplast interfaces. A portable spectroradiometer known as Green-Seeker (Hand Held Optical Sensor Unit, Model 505; NTech Industries, INC., Ukiah, CA, USA) was used to measure NDVI [30].

*Land Surface Temperature.* Land surface temperature (LST) is a good indicator of the energy balance at the earth’s surface and the so-called greenhouse effect, because it is one of the key parameters in the physics of land surface processes on a regional, as well as a global, scale [16]. LST combines the results of surface-atmosphere interactions and energy fluxes between the atmosphere and the ground.

### Sand Fly Collection

**Figure 2:**
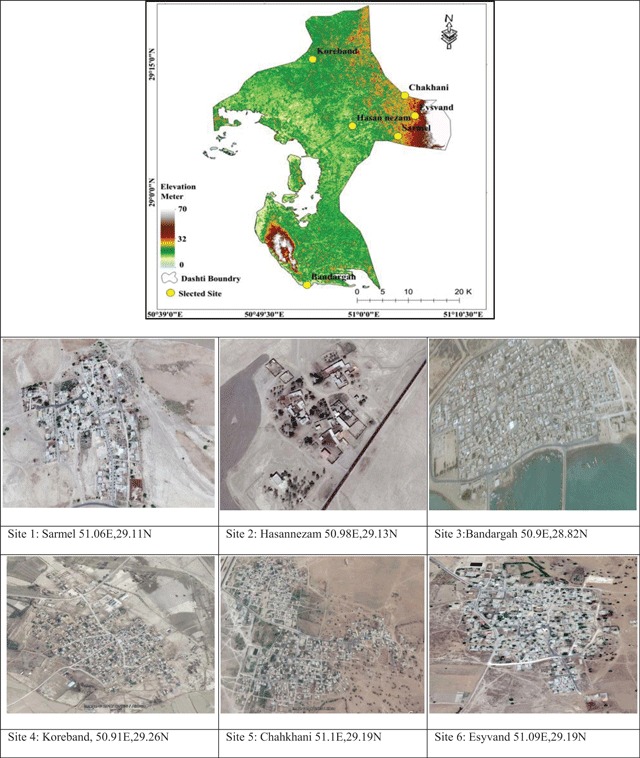
Six rural sites in Bushehr County selected for sand fly collection from January to December, 2016.

Six village sites within Bushehr County were selected for this study (Figure 2). Sarmel 51.06E,29.11N, Hasannezam 50.98E,29.13N, Bandargah 50.9E,28.82N, Koreband, 50.91E,29.26N, Chahkhani 51.1E,29.19N, and Esyvand 51.09E,29.19N. In this study, the sand flies were collected using HP light traps [31]. On nights moonlight could affect the HP light traps, we attached an additional small LED light to increase sand fly attraction and to reduce the moonlight effect.

Monthly all-night landing catches of sand flies were made in the villages for 12 consecutive nights (2 successive nights in each site) from January to December 2016 during one 10-hour collection period from 8 PM to 6 AM. HP light collectors changed positions each night to prevent any bias.

### Statistical Analysis

We examined the spatial association between environmental factors and monthly Phlebotomus sand fly abundance using the Pearson product-moment correlation coefficient, which measures the direction and strength of association between variables. Pearson’s correlation coefficient when applied to a population is commonly represented by following formula:

2 {\rho _{X\,Y}} = \frac{{Cov\left({X,\;Y} \right)}}{{\sigma X\;\sigma Y}}

**where:**

- σX is standard deviation of X
- σY is standard deviation of Y

A 95% confidence level (p = 0.05) determines the significance of correlation coefficients. The relationships between monthly Phlebotomus sand fly abundance as an independent variable and each environmental explanatory variable were analyzed using scatterplots. Finally, multivariate regression models were developed to determine the association between environmental factors and the abundance of caught Phlebotomus sand flies at the 95% level (p = 0.05).

## Results

**Figure 3:**
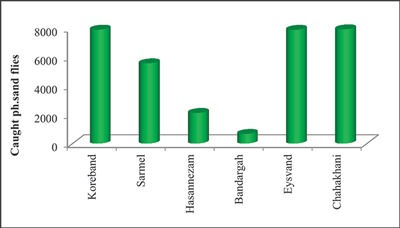
Total caught Ph. sand flies in each selected site.

Spatial distribution of the number of sand flies caught from the mentioned sampling sites is presented in Figure 3. As shown in this figure, the highest number of sand flies caught was at Chah Khani. Of the 32,087 sand flies caught, 7,930 were caught on this site. Koreband and Eysvand had the second and third highest number of sand flies caught at 7,904 and 7,892, respectively. Bandargah had the fewest number of sand flies caught at 669.

Table 2 exhibits the spatial correlation of the sand flies (SF) caught in Bushehr Province in relation to environmental factors, such as daytime and nighttime land surface temperature (MOD13C3), mean, minimum and maximum air temperature (Celsius), radiation (Watt per square meter), precipitation (mm), and vegetation index. As shown in this table, the spatial correlation between the sand flies caught in Bushehr Province with the all environmental factors is positive and direct, except for minimum temperature. In other words, except for the minimum temperature, other variables have a direct linear relationship with the number of caught sand flies. The results of spatial correlation analysis revealed that, in general, the number of sand flies caught has the highest correlation with maximum and mean air temperature, which are equal to 0.81 and 0.89, respectively. These correlation rates, absolutely significant at a confidence level of 0.95, means that the number of sand flies will be significantly higher in parts of the region where the mean and maximum temperature are higher. The correlation between monthly numbers of sand flies caught with the monthly minimum air temperature is equal to 0.31, which means there is an insignificant relationship between the number of sand flies and this variable. Also, the correlation of precipitation with the number of sand flies caught is 0.63, which indicates that precipitation plays a direct role in increasing the sand fly population in the studied area. The correlation between the frequency of sand flies and vegetation index and radiation rate is 0.44 and 0.71, respectively, which is significant at the confidence level of 0.95 (p = 0.05). Nighttime land surface temperature has a correlation of 0.55 with the frequency of sand flies.

**Table 2:** The spatial correlation of sand flies caught and the environmental components.

	Spatial correlation	Sig.

Tmin	0.31	0.07
Tmax	0.1	4 × 10^–8^
Tmean	0.89	4 × 10^–7^
Rain	0.63	32 × 10^–5^
Radiation	0.71	28 × 10^–5^
NDVI	0.44	0.03
DLS	0.62	32 × 10^–5^
NLST	0.53	0.002

**Figure 4:**
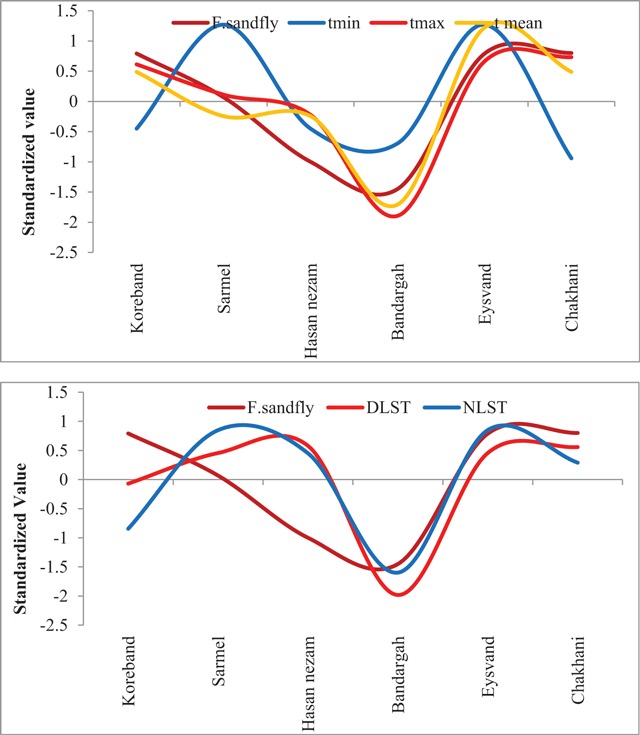
Spatial association among thermal factors and annual caught Ph. sand flies.

In Figure 4, the spatial distribution of thermal parameters, including the mean, minimum and maximum air temperature, and monthly frequency of sand flies in the six sites are presented. As can be seen, with the general increase in mean and maximum air temperature in Eyswand, the frequency of sand flies caught has also increased. In Chah Khani, Hasan nezam, and Bandargah, with decreasing thermal factors (mean and maximum air temperature), the number of sand flies caught was also reduced. In the Sarmel and Koreband, changes in the mean and maximum temperature are directly related and almost consisten with the number of sand flies caught. But in terms of the minimum temperature, as mentioned, a direct and linear relationship with the number of sand flies caught was not observed. In the six study sites, despite the increase in the minimum air temperature, the number of sand flies caught reduced. On an annual scale, with a general increase in the two thermal factors of nighttime and daytime land surface temperature, sites with higher temperatures have the higher number of sand flies (total number of sand flies caught). The spatial correlation of nighttime and daytime surface temperature with the number of sand flies caught is equal to 0.53 and 0.62, respectively.

**Figure 5:**
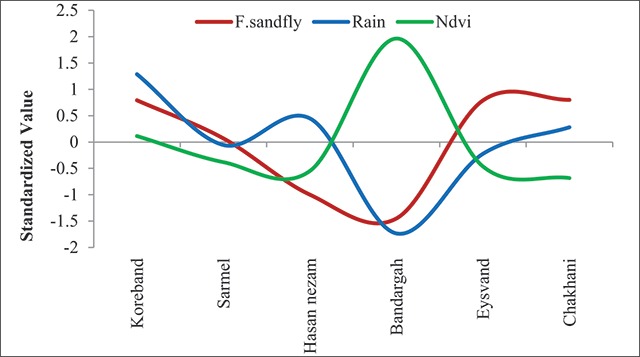
Spatial association between humidity factors and annual caught Ph. sand flies.

The relationship between the total number of sand flies caught and the humidity components of the environment (precipitation and vegetation) is presented in the standardized chart of Figure 5. As seen in the chart, the lowest vegetation cover was observed at the Chah Khani sampling site and the highest number of sand flies caught during the year was also related to this site. The lowest number of sand flies were caught at the Bandargah sampling site, which was richer in terms of vegetation cover than the other sites. The spatial correlation of these two variables was 0.44 in the studied area. On an annual scale, the higher overall number of sand flies (the total number of sand flies caught) was observed in the sites with a higher overall precipitation. The spatial correlation of these two variables was 0.63 in the study area.

According to the correlation matrix, the environmental thermal variables showed a high and significant correlation with the number of sand flies. It should be mentioned that this indicator only shows the direction and magnitude of the relationship and does not estimate the covariation of the dependent variable (the number of caught sand flies) in relation to the independent variable (environmental factors). Furthermore, for modeling and estimating the number of sand flies on the basis of environmental factors, a multivariate linear model was developed. The results of the variance inflation factor (VIF) and tolerance showed the four variables, including maximum and minimum temperature, and nighttime and daytime surface temperature, had a VIF index higher than the limit (a VIF index higher than 10 is considered to be the limit), which indicates these independent variables are highly correlated and there is not the possibility of simultaneous use of them in a multivariable estimator model. Therefore, they were not used for modeling, and the estimation model was developed solely based on four variables: monthly mean temperature, vegetation, radiation, and precipitation. The specifications of this multivariate estimator are presented in Table 3. As can be seen, the amount of changes in the number of sand flies caught in relation to the change in each of the independent variables is presented (Unstandardized Coefficients). Based on the standardized coefficients of the estimation model, the mean air temperature is one of the most important factors determining the monthly population of sand flies.

**Table 3:** The multivariate regression model developed for estimating the number of sand flies caught.

Model		Unstandardized Coefficients		Standardized Coefficients	t	Sig.	Collinearity Statistics	
	
		B	Std. Error	Beta			Tolerance	VIF

1	(Constant)	120258.235	1532694.033		0.078	0.950		
	ndvi	7976.595	43243.444	0.139	0.184	0.884	0.265	3.772
	tmean	20414.181	17059.148	0.865	1.197	0.443	0.287	3.489
	rain	79.286	309.037	0.146	0.257	0.840	0.460	2.176
	rad	–34.795	71.932	–0.208	–0.484	0.713	0.807	1.239

Dependent Variable: fs.

The validation statistic of the model is presented in Table 4. As can be seen, the model based on four independent variables, including mean air temperature, radiation, precipitation, and vegetation, explains about 0.85 of the monthly variance of the caught sand flies population.

**Table 4:** Statistical evaluation of developed linear model for prediction of Ph. sand flies population in Bushehr.

Model	R	R Square	Adjusted R Square	Std. Error of the Estimate	Durbin-Watson

1	0.922^a^	0.850	0.252	2790.12823	2.161

## Discussion

Cutaneous leishmaniasis is one of the six major diseases in tropical regions [1,2,3]. The incidence of this disease is evident in 88 countries (72 of which are developing countries) across all continents, except Australia. Annually, between 1 million and 1.5 million people worldwide are diagnosed with cutaneous leishmaniasis, and nearly 350 million people are at risk for this disease [4,5,6]. Of the number of cases, 90% are from Afghanistan, Algeria, Brazil, Iran, Peru, Saudi Arabia, and Syria, with the highest incidence rates among Iran and Saudi Arabia [7,8]. This disease is one of the most important and common diseases in Iran [2].

**Figure 6:**
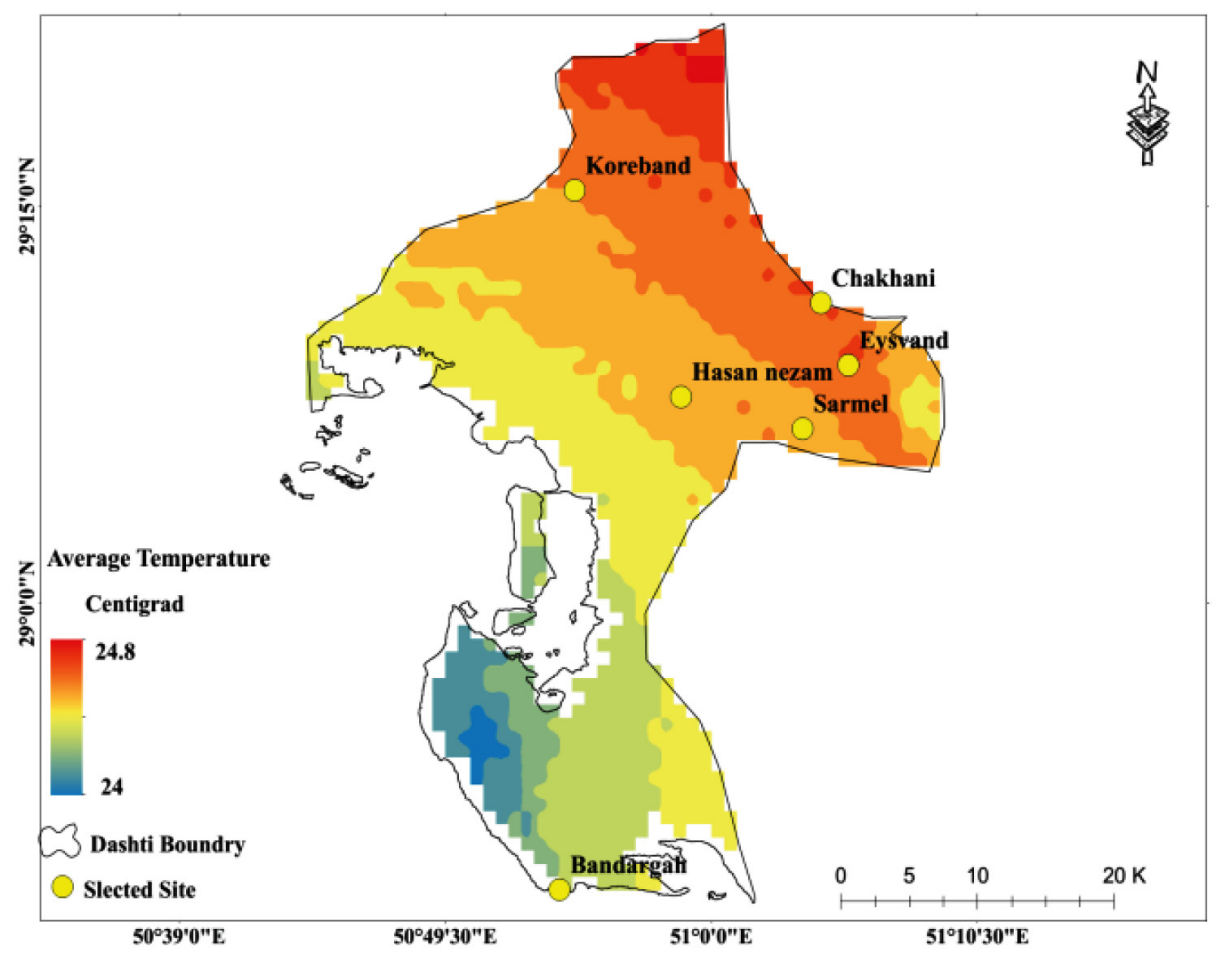
Mean annual temperature as one of the predictors of the Ph. sand fly population in Bushehr Province.

**Figure 7:**
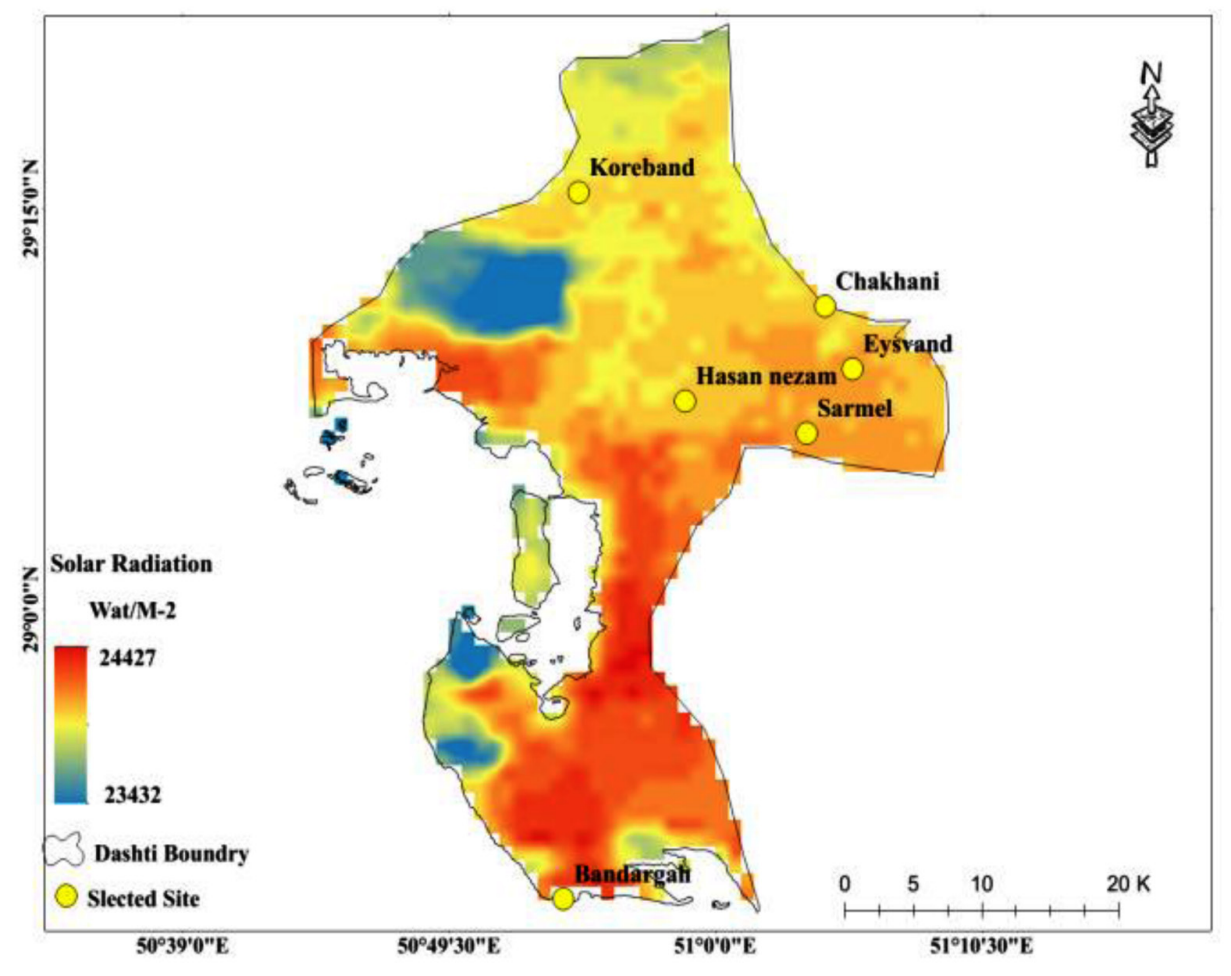
Mean annual solar radiation as one of the predictors of the Ph. sand fly population in Bushehr Province.

**Figure 8:**
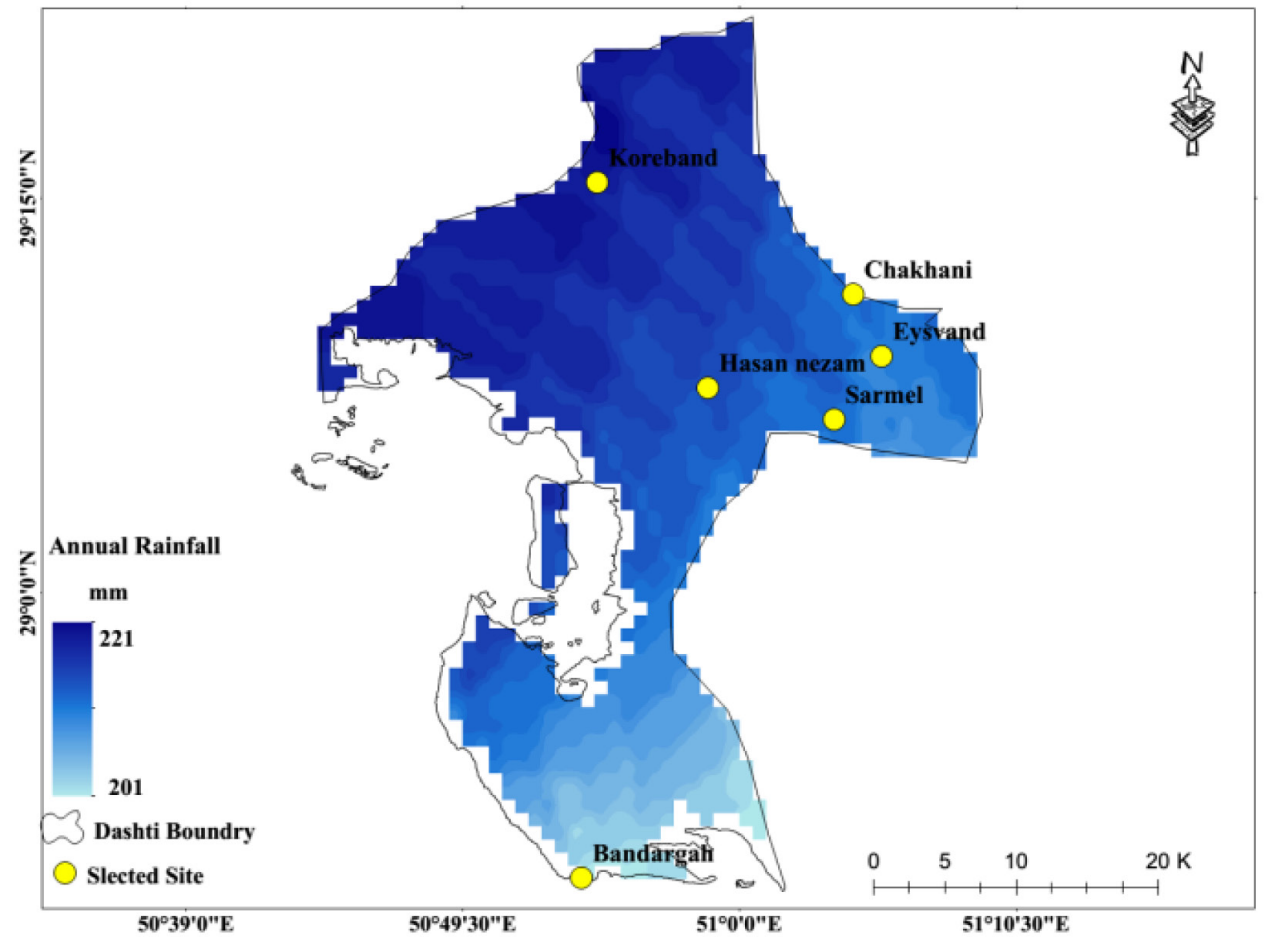
Mean annual rainfall as one of the predictors of the Ph. sand fly population in Bushehr Province.

**Figure 9:**
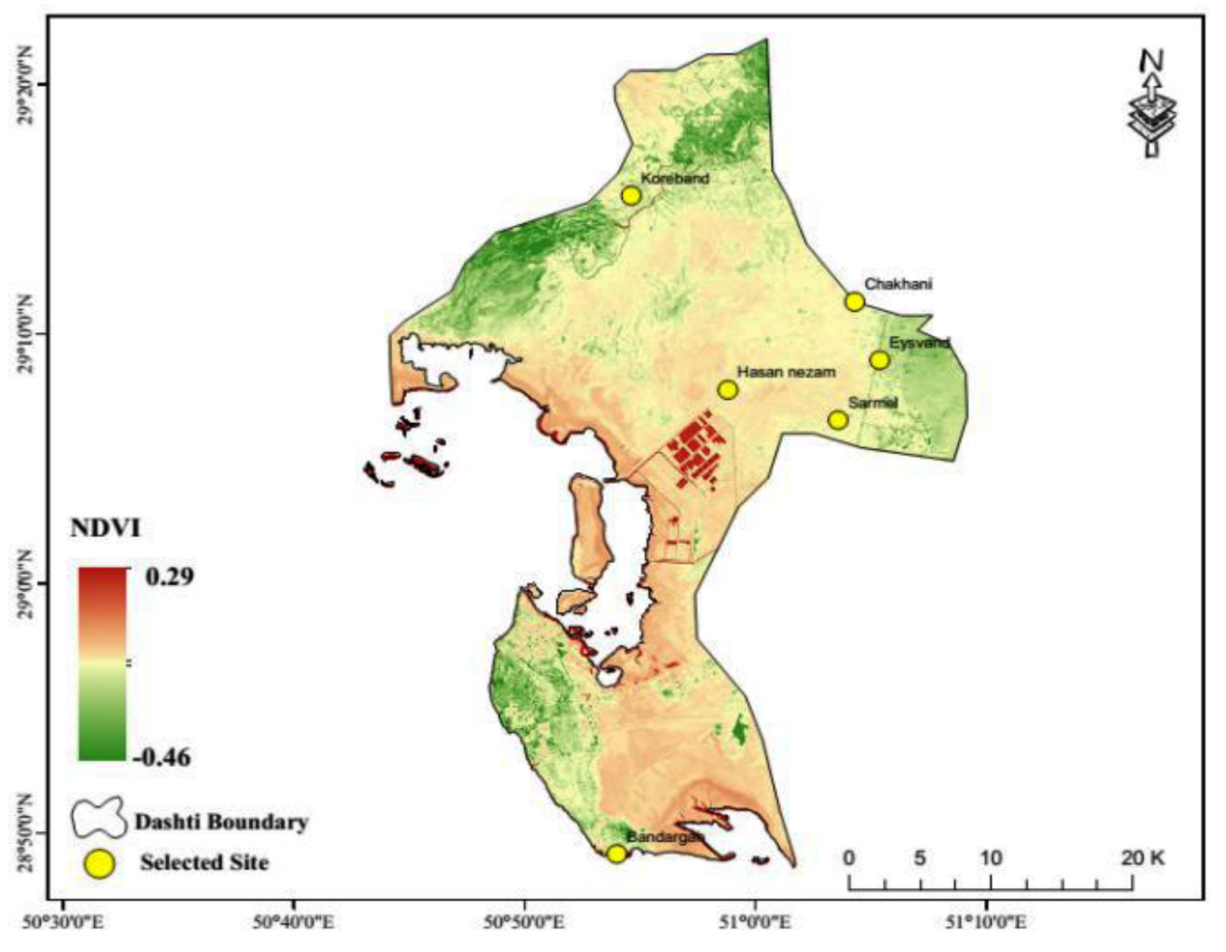
Mean NDVI as one of the predictors of the Ph. sand fly population in Bushehr Province.

The disease has three basic components (Parasite-Reservoir-Vector). The climatic conditions control the incidence of the disease by influencing these components in each region. In Iran, most of the studies on cutaneous leishmaniasis have been conducted by the medical department, and most of them have had a treatment approach. Therefore, the underlying factors that affect the temporal and spatial dynamics of the disease carriers have been less considered. In this study, the spatial frequency of the sand flies caught and their relation with environmental factors, such as temperature, precipitation, vegetation, surface temperature, and radiation were investigated. Spatial distribution of each of the environmental factors involved in modeling is presented in Figures 6, 7, 8, 9.

According to the model, change in temperature was the first lever to control the demographic changes of sand flies. The spatial distribution of temperature is shown in Figure 6. Based on the developed model, as temperature increased by 1°C, the total number of sand flies caught increased by 20,414 over the studied area. The 15-year average range of temperatures between the coldest and hottest parts of the region is equal to 0.8°C. In the northern regions of the study area, the temperature is 24.8°C, while the temperature in the southern regions decreases to 24°C.

According to the model, radiation was the second lever controlling the demographic changes of the sand flies. The spatial distribution of the radiation is shown in Figure 7. As the radiation rate increased 1 W/m^2^, the total number of sand flies caught decreased by 34 over the studied area. In the southern region of the study area, radiation was higher.

The spatial distribution of precipitation in the study area is shown in Figure 8. According to the results obtained from the model, precipitation variation is the third most influential climatic element in the sand fly population. The total number of sand flies caught increased by 79 as a result of the increase in each millimeter of precipitation. According to Figure 8, the northern regions of the study area experience more precipitation (221 mm), while in the southern regions, precipitation drops 20 mm and reaches 201 mm.

The spatial distribution of vegetation cover in the study area is shown in Figure 9. According to the results of the model, vegetation cover was the fourth most influential climatic element in the sand fly population. With the increase of each unit of vegetation cover, the total number of sand flies caught increased by 7,976. According to Figure 9, the northern region of the study area has more vegetation cover than other areas, while there is very little vegetation cover in the central areas.

**Figure 10:**
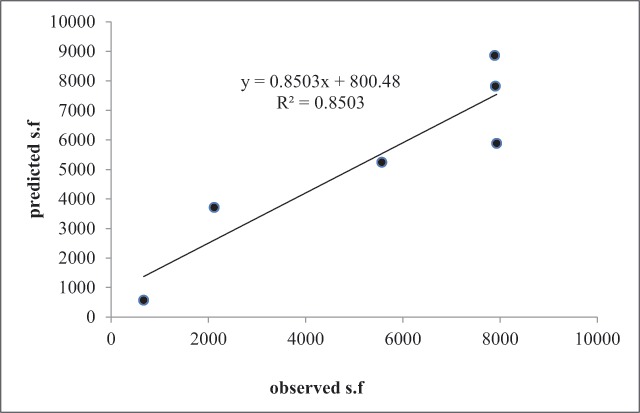
Predicted Ph. sand flies vs observed caught sand flies.

Figure 10 shows the estimated and observed values of sand fly population caught. The model presented in this study is a climate model, which explains distribution of the sand flies caught using four climatic parameters, including mean air temperature, precipitation, radiation, and vegetation index. As can be seen, based on the proposed models, environmental factors explain 85% of the temporal variance of the sand fly population caught.

## Conclusion

Environmental and climatological factors are amongt the most important factors in controlling carriers and reservoirs of many infectious diseases. Because of the high cost of treating these diseases (malaria and cutaneous leishmaniasis) and because many of the countries involved are developing countries, treating these diseases is one of the main obstacles facing these countries. Knowing the environmental and climatological factors that influence the temporal and spatial dynamics of the disease carriers and their reservoirs, and consequently the incidence of the disease, the disease can be effectively controlled.

The main objective of this research was to analyze the relationship between the spatial distribution of caught sand flies and climatic conditions in Bushehr Province. The results indicated that all of the thermal factors of the environment (mean and maximum temperature, radiation, night and day time surface temperature, precipitation and vegetation cover), except the minimum temperature, have a direct and strong correlation with the number of sand flies caught in the studied areas. The results of the estimation model indicate that these environmental estimators, including four climatic elements (mean air temperature, precipitation, radiation and vegetation index), in a linear estimation model can explain 0.85 of the spatial variations of the sand flies caught.
